# Study protocol for a single-blind, parallel-group, randomized, controlled superiority trial of intensive versus weekly delivered prolonged exposure for adults with post-traumatic stress disorder

**DOI:** 10.1186/s13063-024-08218-2

**Published:** 2024-06-12

**Authors:** Maria Bragesjö, Brooke Fina, Ekaterina Ivanova, Volen Z Ivanov, Christian Rück

**Affiliations:** 1 Department of Clinical Neuroscience, Centre for Psychiatry Research, Karolinska Institutet & Stockholm Health Care Services, Region Stockholm, Stockholm, Sweden; 2 Department of Psychiatry and Behavioral Sciences, Division of Behavioral Medicine, School of Medicine, University of Texas Health Science Center at San Antonio, San Antonio, USA

**Keywords:** Post-traumatic stress disorder, Trauma-focused cognitive behavior therapy, Intensive treatment, Massed treatment, Prolonged exposure

## Abstract

**Background:**

Prolonged exposure (PE) therapy is widely recognized as an effective treatment for post-traumatic stress disorder (PTSD) and is often considered one of the primary options for addressing this condition. Nevertheless, a significant proportion of patients (30–51%) fail to demonstrate clinically significant symptom changes. One of the reasons is that a high proportion of patients drop out from treatment, which often lasts for a minimum of 3–4 months. Hence, there is an urgent need for PTSD treatments that can be delivered to decrease dropout rates. A more intensive PE treatment approach has been suggested to decrease dropout rates and in addition achieve faster recovery rates and has shown promising effects on reducing PTSD symptoms but needs to be tested against firsthand treatment.

**Methods:**

This single-blind, randomized controlled trial (*N* = 140) will compare an intensive delivery format of prolonged exposure (iPE) against standard weekly delivered sessions of PE. The primary outcome is change on the Clinician-Administered PTSD Scale for DSM-5 (CAPS-5). Secondary outcomes include self-rated measures of symptoms of PTSD and complex PTSD, depression and quality of life, speed of recovery, cost effectiveness, dropout rates, and adverse events.

**Discussion:**

This study will be the first to compare iPE with first-line treatment in a psychiatric outpatient setting. One of the key strengths of this study lies in its implementation within a clinical setting and the broad eligibility criteria. Additionally, the utilization of gold-standard assessment measures ensures the accuracy and reliability of the outcomes. However, several potential challenges may arise during the study’s execution. These challenges may include difficulties in participant recruitment, ensuring adequate participant retention, adherence to the treatment protocol, and maintaining therapist retention mostly due to recruitment taking place at one single clinic.

**Trial registration number:**

Clinicaltrials.gov NCT05934175. Registered on June 6, 2023. Open Science Framework (OSF) https://osf.io/7qsb3. Registered on September 2, 2023.

## Administrative information

**Table:** 

Title	Study protocol for a single-blind, parallel-group, randomized, controlled superiority trial of intensive versus weekly delivered prolonged exposure for adults with post-traumatic stress disorder
Trial registration {2a and 2b}	Clinicaltrials.gov NCT05934175; Open Science Framework (OSF) https://osf.io/7qsb3
Protocol version {3}	2023–11-08 version 2.0
Funding {4}	This study is funded by FORTE (2023–01220), CIMED (FoUI-960456), and Region Stockholm (ALF project FoUI-972375 and Postdoc FoUI-987469)
Author details {5a}	^1^Centre for Psychiatry Research, Department of Clinical Neuroscience, Karolinska Institutet & Stockholm Health Care Services, Region Stockholm, Stockholm, Sweden. ^2^Division of Behavioral Medicine, Department of Psychiatry and Behavioral Sciences, School of Medicine, University of Texas Health Science Center at San Antonio, San Antonio, USA
Name and contact information for the trial sponsor {5b}	Study sponsor: Region Stockholm, Stockholm Sweden. Contact: managing director Lina Martinsson; lina.martinsson@regionstockholm.se; Psykiatri Sydväst, M 58 Huddinge sjukhusområde, 141 86 Stockholm; 08–123 800 00
Role of sponsor {5c}	The funders had no role in study design, data collection and analysis, decision to publish, or preparation of the manuscript

## Background {6a}

Post-traumatic stress disorder (PTSD) is characterized by distressing symptoms such as intrusions from the traumatic event, avoidance of trauma reminders, cognitive and mood changes, and arousal symptoms. Its onset is associated with considerable functional and work impairment and psychiatric and medical comorbidity [1–4].

Individual trauma-focused CBT, such as prolonged exposure (PE), is considered a first-line treatment for PTSD in clinical guidelines. Prolonged exposure is typically delivered weekly during a period of 3 to 4 months, which creates a large time window for disruption from unexpected life events, avoidance, or loss of motivation. Despite being a first-line treatment, the response rate for PTSD treatments like PE is only around 60% [5], partly due to high dropout rates, which can reach up to 52% [6]. Patients may hesitate to engage in exposure techniques due to fear, and initial treatment phases might even worsen symptoms before improvement occurs. Intensive treatment formats, characterized by close and continuous therapist-patient contact, offer advantages over standard weekly sessions. They provide more opportunities to address avoidance behaviors, low self-efficacy, and motivational issues. Additionally, the condensed timeframe of intensive treatment may reduce interference from unexpected life events and decrease dropout rates. In summary, intensive treatment approaches may offer a solution to the limitations of standard PTSD therapies, potentially leading to quicker recovery and better resource utilization.

According to a recent systematic review, intensive treatments for PTSD demonstrate potential benefits, such as improved treatment response, faster recovery, and reduced treatment dropout. The review found a large impact on the reduction of PTSD symptoms (weighted mean effect *d* = 1.57, 95% CI [1.24, 1.91]) and notably high rates of treatment completion (5.5% pooled dropout rate across studies) [7]. Nevertheless, the findings should be interpreted with caution, as over 80% of the studies were uncontrolled. Since this review, a randomized clinical trial involving US military personnel and veterans treated with massed or intensive outpatient formats of prolonged exposure therapy resulted in clinically significant reductions in clinician-assessed PTSD symptoms with 50% of participants reaching PTSD diagnostic remission at the 6-month follow-up [8]. Thus, intensive treatment may be an effective treatment format, but its efficacy and cost-effectiveness have not yet been investigated in a psychiatric setting as a head-to-head comparison with first-line treatment for PTSD in individual weekly sessions of prolonged exposure.

In this single-blind, superiority, randomized controlled trial, we will evaluate the efficacy of intensive prolonged exposure compared to first-line treatment (weekly delivered prolonged exposure) for adults with PTSD in a Swedish psychiatric setting. If effective, intensive prolonged exposure could be a welcome addition to available evidence-based options for this target group and reduce dropout.

## Objectives {7}

The main objectives are to investigate the efficacy of intensive exposure-based treatment (iPE) for PTSD in a randomized controlled trial (*N* = 140) comparing iPE with weekly delivered prolonged exposure. The trial will encompass an assessment of health-economic factors from two distinct viewpoints: one from the standpoint of the treatment provider (comprising direct expenses such as healthcare personnel costs) and the other from a more extensive societal standpoint (encompassing indirect expenses such as sick leave). Additionally, we will measure treatment-related transformational processes and factors that can influence treatment results.

The two types of treatment formats will be compared 1 month after treatment completion in each respective treatment arm (primary endpoint) and at the 6-month and 12-month follow-ups regarding clinician-rated PTSD symptom severity by assessors masked to treatment allocation. In addition, comparisons in relation to self-rated symptoms of PTSD and complex PTSD, depression and quality of life, recovery rate, response and remission rates, number of dropouts, cost-effectiveness, adverse events, treatment mediators and moderators, credibility, satisfaction with treatment, and perceived alliance with the therapist will be made. Due to its shorter format, we expect the intensive treatment format to provide a faster response than first-line treatment.

The research questions to be answered within the project are as follows:

1. Is iPE more efficacious than weekly delivered sessions of prolonged exposure regarding reduction of blinded assessor rated PTSD symptom severity 1-month posttreatment in each respective treatment arm?
2. Is iPE more cost-effective than weekly delivered sessions of prolonged exposure at 1 month, 6 months, and 12 months posttreatment?
3. Does iPE lead to less dropout than weekly delivered sessions of prolonged exposure up to 1 month posttreatment?
4. Does iPE lead to more adverse events than weekly delivered sessions of prolonged exposure up to 12 months posttreatment?
5. Does iPE lead to a faster recovery rate than weekly delivered sessions of prolonged exposure up to 12 months posttreatment?
6. Is iPE more efficacious than weekly delivered sessions of prolonged exposure regarding the reduction of self-rated depression and increased quality of life at 1 month, 6 months, and 12 months posttreatment?
7. What is the mechanism of change of prolonged exposure? Does the mechanism of change in iPE and weekly delivered sessions differ?
8. What are the moderators of the treatment effect?
9. Are the therapeutic gains of iPE maintained at long-term follow-up (6 and 12 months after treatment)?

## Trial design {8}

**Fig. 1:**
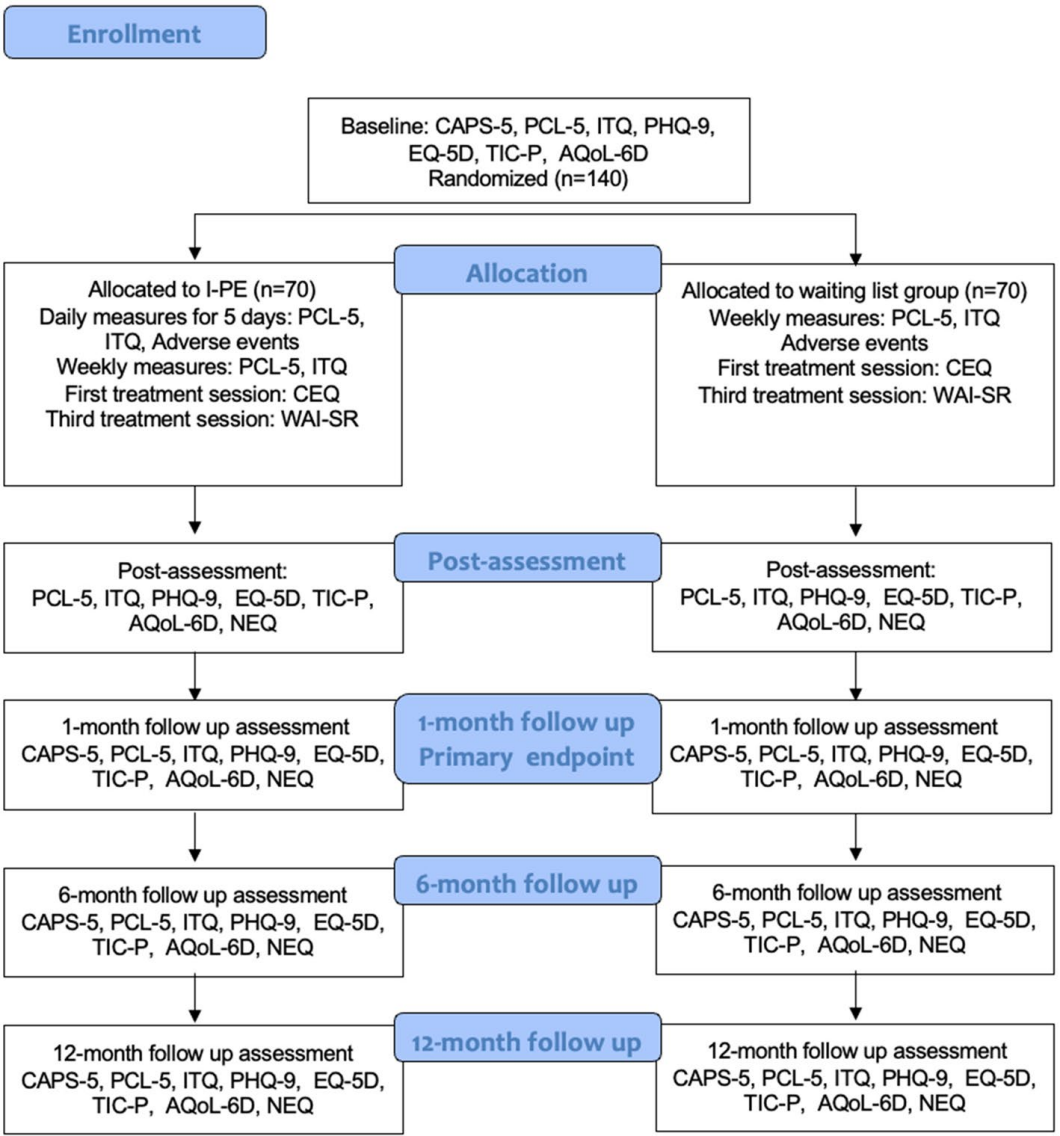
Flow chart of the trial

The trial uses a single-blind (assessors masked to treatment allocation), randomized (1:1), controlled, parallel-group, superiority design comparing intensive prolonged exposure and first-line treatment for adults with PTSD residing in Sweden. We aim to recruit 140 participants who will be assessed before treatment start, during treatment, 1 month after treatment completion in each respective treatment arm (primary endpoint), and at follow-ups 6 and 12 months after treatment completion. The flow chart of the trial is shown in Fig. 1.

The trial was approved by the Swedish Ethical Review Authority (ID: 2023–02329-01) and was preregistered on Clinicaltrials.gov and Open Science Framework (osf.io) before data collection started. Data will be reported in accordance with The Consolidated Standards of Reporting Trials statement for nonpharmacological trials (CONSORT) and Consolidated Health Economic Evaluation Reporting Standards (CHEERS) irrespective of outcome. All study personnel will be trained in Good Clinical Practice (GCP). Quality and safety aspects of the trial will be monitored by an external part (A + Science).

## Methods

### Study setting {9}

The study will be conducted at an outpatient psychiatric clinic in Stockholm, Sweden, which is funded by public resources.

### Eligibility criteria {10}

To ensure the study’s external validity, we will adopt broad inclusive eligibility criteria. Participants presenting with comorbidities such as neuropsychiatric disorders, depression, and substance abuse will be evaluated for inclusion based on meeting all other specific inclusion/exclusion criteria. The primary diagnosis for inclusion will be PTSD.

Please see Table 1 for a comprehensive outline of the inclusion and exclusion criteria.

**Table 1:** Inclusion and exclusion criteria

Inclusion criteria	≥ 18 years of age
	Primary diagnosis of PTSD according to DSM-5
	Signed informed consent
	Able to attend either iPE or 15 weekly sessions of PE
	Being fluent in Swedish
Exclusion criteria	Initiation or adjustment of any psychotropic medication within the last 4 weeks prior to inclusion
	Serious mental health symptoms, such as mania, psychosis, alcohol, or substance use disorders or current suicide risk warranting immediate clinical attention
	Ongoing evidence-based trauma-focused psychological treatment
	Ongoing trauma-related threat (e.g., living with a violent spouse)

### Informed consent {26a}

Informed consent will be obtained during the registration in the secure digital platform requiring two-step authentication.

### Additional consent provisions for collection and use of participant data and biological specimens {26b}

This trial does not involve collecting biological specimens for storage.

### Interventions

#### Explanation for the choice of comparators {6b}

The trial will compare prolonged exposure to weekly delivered sessions, which is considered first-line treatment [9].

### Intervention description {11a}

#### Intensive treatment (iPE)

The intensive treatment adheres to the original treatment protocol for prolonged exposure [10] with three major exceptions. First, treatment is delivered for five consecutive days followed by three 60-min individual booster sessions dispersed 1, 2, and 4 weeks afterwards. Second, treatment combines individual and group formats. Each patient will receive nine individual sessions focused on confronting the memory of the traumatic event (imaginal exposure and processing) and five group sessions with psychoeducation, breathing retraining, overall rationales, and preparing work with confronting trauma-related situations in the patient’s daily life (in vivo exposure). Starting from day 2, the group sessions shift their focus to the formulation of individually tailored designed exercises for individual implementation during these sessions. Third, there is no assigned homework between treatment days; instead, time is allocated between daily sessions for working on exposure exercises.

The treatment will be delivered for an average of 7 h of treatment each day for five consecutive days. Three individual 60-min booster sessions will be conducted, which will include imaginal exposure, follow-up on in vivo exposure exercises, a comprehensive summary of the treatment and lessons learned, as well as discussions on relapse prevention and planning for continued self-practice. These booster sessions will be dispersed 1, 2, and 4 weeks after the intensive treatment week. The total treatment period is 5 weeks, which encompasses the intensive treatment and booster sessions.

Table 2 shows a typical treatment schedule for the iPE week.

**Table 2:** A typical treatment schedule for the iPE week

Monday	Tuesday	Wednesday	Thursday	Friday
**INTRO MEETING** 9:00 – 9:30	**IMAGINAL EXPOSURE SESSION 2** 8:30 – 9:30	**IMAGINAL EXPOSURE SESSION 4** 8:30 – 9:30	**IMAGINAL EXPOSURE SESSION 6** 8:30 – 9:30	**IMAGINAL EXPOSURE SESSION 8** 8:30 – 9:30
**GROUP SESSION 1 PSYCHOEDUCATION, OVERALL RATIONALE, PREPARATION IN VIVO** 9:30 – 11:00	Break 9:30 – 10:00	Break 9:30 – 10:00	Break 9:30 – 10:00	Break 9:30 – 10:00
	**OWN WORK** 10:00 – 11:00	**OWN WORK** 10:00 – 11:00	**OWN WORK** 10:00 – 11:00	**OWN WORK** 10:00 – 11:00
**LUNCH** 11:00 – 12:00	**LUNCH** 11:00 – 12:00	**LUNCH** 11:00 – 12:00	**LUNCH** 11:00 – 12:00	**LUNCH** 11:00 – 12:00
**WALK OUTSIDE** 12:00 – 12:30	**GROUP SESSION 2** **IN-VIVO EXPOSURE** 12:00–13:45	**GROUP SESSION 3** **IN-VIVO EXPOSURE** 12:00–13:45	**GROUP SESSION 4** **IN-VIVO EXPOSURE** 12:00–13:45	**GROUP SESSION 5** **IN-VIVO EXPOSURE** 12:00–13:45
Break 12:30 – 13:00				
**IMAGINAL EXPOSURE SESSION 1** 13:00 – 14:00				
	Break 13:45 – 14:00	Break 13:45 – 14:00	Break 13:45 – 14:00	Break 13:45 – 14:00
Break 14:00 – 14:15	**IMAGINAL EXPOSURE SESSION 3** 14:00 – 15:00	**IMAGINAL EXPOSURE SESSION 5** 14:00 – 15:00	**IMAGINAL EXPOSURE SESSION 7** 14:00 – 15:00	**IMAGINAL EXPOSURE SESSION 9** 14:00 – 15:00
**OWN WORK** 14:15 – 15:15				
	Break 15:00 – 15:15	Break 15:00 – 15:15	Break 15:00 – 15:15	Break 15:00 – 15:15
	**OWN WORK** 15:15 – 16:15	**OWN WORK** 15:15 – 16:15	**OWN WORK** 15:15 – 16:15	**OWN WORK** 15:15 – 16:15

#### Weekly prolonged exposure

Patients will receive 15 weekly 90-min sessions of individual prolonged exposure for PTSD according to the standard treatment delivery protocol. In session 1, a comprehensive overview of treatment rationale will be provided, along with the selection of the index trauma, which will be the focus of imaginal exposure and breathing retraining. Session 2 will include psychoeducation on trauma and PTSD, along with a rationale for in vivo exposure and the construction of an in vivo hierarchy. In session 3, imaginal exposure including processing for the trauma will commence following an overall rationale of this core treatment component. Starting from session 4 and continuing through session 14, participants will engage in imaginal exposure during the sessions and continue in vivo exposures outside of the therapy sessions. Assigned homework during the treatment weeks will include reading treatment material, practice breathing retraining, listen to recordings of imaginal exposure, and conducting in vivo exercises. Session 15 will include a summary of the treatment and lessons learned, as well as relapse prevention and planning of continued self-practice.

### Criteria for discontinuing or modifying allocated interventions {11b}

Participants have the right to withdraw their consent to participate in the treatment and the trial at any time point. In the event of an increase in suicidal ideation, an assessment will be conducted to determine if a referral to another appropriate resource is necessary.

### Strategies to improve adherence to interventions {11c}

The therapist will contact participants who cancel or do not show up at appointments by phone, and if the participant cannot be reached, send the participant a letter according to established clinical routines.

#### Therapist competence and adherence

The therapists in the trial will be resident psychologists and clinical psychologists ranging in experience from limited to extensive in PE and iPE before the start of the study. Each therapist will provide treatment to an equivalent portion of patients from both treatment groups. The therapists will receive a minimum of 2 days of training in prolonged exposure and in the iPE protocol. Supervision by certified supervisors and trainers in prolonged exposure will be conducted on a weekly basis. During the intensive treatment week, the therapists will receive daily supervision. Audio recordings of the sessions will be made, and for each therapist’s first treated participant in the study, ratings of adherence and competence will be conducted for the sessions containing the central treatment components. These components include the overall rationale of treatment, introduction to in vivo exposure and constructing an in vivo hierarchy, rationale and instructions for imaginal exposure and processing, and rationale and instructions for hot spots and relapse prevention. In addition, 20% of the recordings will be randomly selected and rated for adherence to the protocol. The ratings will be performed by independent supervisors in prolonged exposure using the Therapist Adherence and Competence Rating Scale for prolonged exposure (Nishith P, Resick PA: Adherence and competence rating scale for prolonged exposure treatment, unpublished). Adherence to the study protocol will be rated using checklists developed specifically for each part of the study.

####  Assessor competence

Blinded assessors will undergo at least 1 full day of training in the CAPS-5 administration and scoring prior to conducting clinician-rated assessment and practice on videos of PTSD case examples. Assessors who deviate by more than 10% of the CAPS-5 total score agreed by one of the authors (MB) or score more than one point differently on four or more items will need to repeat the training. To ensure reliability of ratings, assessors will have access to weekly supervision. Furthermore, assessors will be required to engage in the practice with videos demonstrating PTSD case examples twice annually.

### Relevant concomitant care permitted or prohibited during the trial {11d}

Please see the predefined inclusion and exclusion criteria; participants must have maintained a stable dose of any psychotropic medication for a minimum of 4 weeks before entering the study. Additionally, concurrent participation in any other ongoing trauma-focused CBT or Eye Movement Desensitization and Reprocessing therapy is not permitted at the time of inclusion in the study and participants are recommended not to initiative these types of treatment throughout the treatment duration.

### Provisions for posttrial care {30}

Upon the termination of the study, participants identified as requiring additional treatment will receive assistance in locating suitable and appropriate follow-up care.

### Outcomes {12}

Table 3 presents the flow of the recruitment and treatment process and lists clinician-rated and self-rated assessments at the different time points.

**Table 3:** The flow of the recruitment and treatment process

	Enrollment	Allocation	Baseline	Week 1–5	Week 6–15	Posttreatment	1-month follow-up (primary end-point)	6-month follow-up	12-month follow-up
**Enrollment**									
Eligibility screen	X								
Informed consent	X								
Allocation		X							
Treatment									
Weekly PE				X	X				
iPE				X					
**Clinical administered instruments**									
CAPS-5	X						X	X	X
MINI	X								
**Self-rated instruments**									
PCL-5^a^			X	X	X	X	X	X	X
ITQ^a^			X	X	X	X	X	X	X
PHQ-9^b^			X		X	X	X	X	X
EQ-5D^b^			X		X	X	X	X	X
AQoL-6D^b^			X		X	X	X	X	X
TIC-P^b^			X		X	X	X	X	X
NEQ^b^					X	X	X	X	X
CEQ^c^				X					
WAI-SR^d^				X					
Treatment preference	X						X		
SUD-ratings^e^				X	X				

*Abbreviations*: *AQoL-6D* Assessing Quality of Life 6 Dimensions, *CAPS-5* Clinician-Administered PTSD Scale for DSM-5, *CEQ* Credibility/Expectancy Questionnaire, *ITQ* The International Trauma Questionnaire, *MINI* Mini International Neuropsychiatric Interview, *NEQ* Negative Effects Questionnaire, *SUD* Subjective Units of Distress Scale, *TIC-P* Treatment Inventory of Costs in Psychiatric Patients, *PHQ-9* Patient Health Questionnaire, *WAI-SR* Working Alliance Inventory–Short Form Revised

^a^PCL-5 and ITQ are administered daily during the intensive phase of treatment in the I-PE group

^b^PHQ-9, EQ-5D, AqoL-6D, TIC-P, and NEQ are administered immediately after treatment completion, w5 in the I-PE arm and w15 in the weekly delivered PE arm

^c^CEQ is administered after the first individual session in each treatment format

^d^WAI-SR is administered at the third individual session in each treatment format

^e^SUD-ratings are collected each individual treatment session

The primary outcome measure is the gold-standard clinical interview CAPS-5 that assesses PTSD symptom severity and past month diagnosis of PTSD [11] administered at enrollment, 1 month after treatment completion (primary endpoint), and at 6- and 12-month follow-ups. The CAPS-5 total severity score has high internal consistency (Cronbach’s *α* = 0.88) and interrater reliability (intraclass correlation coefficient = 0.91) and good test–retest reliability (intraclass correlation coefficient = 0.78) [11]. In this study, treatment response will be deemed successful if there is a minimum improvement of 10 points on the CAPS-5 scale between baseline and the participant’s last recorded measurement within the baseline to 12-month follow-up period. Remission will be defined as the absence of a PTSD diagnosis and/or a CAPS-5 total symptom severity score below 24, following the criteria set by Blevins et al. [12]. To be categorized as having achieved recovery, participants must maintain their remission status through the subsequent follow-up assessment.

Secondary self-rated outcome measures are the Post-traumatic Stress Disorder Checklist for DSM-5 (PCL-5; [12]) and the International Trauma Questionnaire (ITQ; [13, 14]). These instruments are utilized to capture self-rated symptoms of PTSD and complex PTSD. Treatment response on the PCL-5 will be defined as a reduction of 10 points or more from the baseline score. Remission will be determined by a score below 30 on the PCL-5, as identified in the Swedish version of the scale by Bondjers [15], which is indicative of probable PTSD. To be classified as having achieved recovery, participants must maintain their remission status through the subsequent follow-up assessment.

Additionally, the level of depressive symptoms will be assessed using the Patient Health Questionnaire (PHQ-9; [16, 17]), and quality of life will be assessed using the EQ-5D [18].

The Assessing Quality of Life 6 Dimensions (AQoL-6D; [19]) will be administered to calculate quality-adjusted life-years for cost-utility analysis. The Treatment Inventory of Costs in Psychiatric Patients will be used to collect information on the use of medical resources, medication, social care, absenteeism, and presenteeism (TIC-P; [20]).

The Negative Effects Questionnaire (NEQ; [21]) will be used to capture potential adverse events immediately after treatment completion and the upcoming assessment points. An open-ended question asking for the occurrence of any adverse events will also be administered at each treatment session.

#### Treatment variables

Participants will also be asked to state their treatment format preference pre- and posttreatment on a 7-point Likert-type scale. Data will also be collected on the number of attended sessions for each participant (including any additional time needed, for example, phone calls between sessions) and protocol deviations. Ratings of the patient’s subjective level of distress for each exposure treatment session will also be collected. Treatment credibility and working alliance will be evaluated between the treatment arms with the Credibility/Expectancy Questionnaire (CEQ; [22]) and Working Alliance Inventory–Short Form Revised (WAI-SR; [23]).

A treatment completer is defined as a participant who completed all treatment sessions or recovered early. Early recovery is defined as a participant who demonstrates a positive response after at least 6 treatment sessions involving imaginal exposure, with a minimum of a 10-point reduction on the PCL-5. Both the participant and therapist must mutually agree to terminate the treatment.

Participants who drop out or recover early from the treatment will be encouraged to continue to participate in the study and all the outcome assessment points.

### Participant timeline {13}

Applicants register on an encrypted study platform that uses a two-step authentication where information about the study and the informed consent form can be found. Prospectively eligible applicants will undergo a thorough assessment of eligibility using the MINI, CAPS-5, and a structured suicide risk evaluation assessment. Eligible participants will be invited to participate in the study if the inclusion criteria are met, as stated in Table 1.

### Sample size {14}

A power calculation was conducted to determine the number of participants needed to evaluate intensive treatment compared to weekly delivered sessions. The power analysis for the trial is based on the CAPS-5, where a significant difference in treatment effects between groups would be set to at least 10 points. With an estimated standard deviation of the CAPS-5 of 20, this represents a standardized mean difference in terms of Cohen’s *d* of 0.5 [24]. Given 95% power, 10% data attrition rate, and an alpha level of 0.05, we estimate that we would need a total of 140 participants to find a statistically significant moderate between-group effect of *d* = 0.5 at our primary endpoint (1 month). We also conducted a power analysis to ensure that we would be able to detect our expected differences in dropout rate, a reduction in the dropout rate from a 22% baseline rate to 5% in the intervention group with conditional linear regression, conditional on the four randomization strata arising from the combination of sex (male, female) and trauma onset (childhood, adulthood). The power was estimated with 1000 randomly generated datasets. The following table shows the estimated power with different sample sizes (*n*).

**Table:** 

*n*	Mean (reject)
100	0.759
120	0.820
140	0.878
160	0.925

### Recruitment {15}

The study will recruit participants from a psychiatric outpatient clinic in Stockholm. All clinical personnel at the recruitment site will have access to an information sheet of the study, which they can present and discuss with patients. If this will not be enough to recruit participants, self-referrals will be made available through a dedicated website, and advertisements will be placed on social media with information about the study.

### Assignment of interventions: allocation

#### Sequence generation {16a}

After the baseline assessment, participants will be randomly assigned in a consecutive manner, using a 1:1 ratio for the randomization process using block randomization. Participants will be stratified according to trauma type (exposure in childhood or as an adult) and gender. The Karolinska Trial Alliance has programmed the randomization sequence in a digital system. The system generates a unique allocation certificate with participant ID, allocation, personnel ID, and time stamp.

#### Concealment mechanism {16b}

The allocation sequence is completely concealed from all study personnel.

#### Implementation {16c}

The project leader will assign participants to interventions.

### Assignment of interventions: blinding

#### Who will be blinded {17a}

The therapists and the participants in the trial cannot be blinded to treatment allocation due to the complex nature of the treatment, but group allocation will be masked to the assessors up to completion of the 12-month follow-up. Participants will receive explicit instructions not to discuss their treatment allocation with the assessor. If a participant still unintentionally discloses their treatment allocation, another blind assessor will reassess the diagnosis and symptom severity using recordings of the CAPS-5 interviews. The participant will also be assigned another assessor at subsequent follow-ups. After each assessment, the assessors will be asked to guess the participants’ allocation, and answers will be compared to chance at the end of the study to measure blinding integrity. Data managers, primary outcome assessors and trial statisticians will be blinded to group allocation until the final data analyses are completed.

#### Procedure for unblinding if needed {17b}

Emergency unblinding will be implemented when deemed clinically necessary, for example, in situations where treatment decisions demand knowledge of the allocated intervention or when there is an unexpected occurrence of a serious adverse event.

### Data collection and management

#### Plans for assessment and collection of outcomes {18a}

Participants will complete baseline assessments, measures at each individual treatment session, immediately after treatment completion and at the 1-month, 6-month, and 12-month follow-ups. All data are collected in BASS, a secure digital platform developed by Karolinska Institutet.

#### Plans to promote participant retention and complete follow-up {18b}

The digital platform is equipped to send out automatic reminders if assessments are overdue. The assessors will contact participants who cancel or do not show up at appointments by phone, and if no contact is made, send the participant a letter according to established clinical routines.

### Data management {19}

Participants will complete online questionnaires through the online platform BASS, hosted on Karolinska Institute’s secure computing facilities, with data backup handled by the same institute. The platform ensures additional security through two-factor authentication. The digital data, including audio files, are stored on encrypted servers, requiring a VPN connection, a personal service card, and/or two-step authentication in accordance with local data management guidelines. The analog data gathered during the study are securely stored within a locked file cabinet. Access to this cabinet is limited to the principal investigator and the study coordinator, who hold the key. Additionally, entry to the archive room corridor necessitates a personal service card, and access to the room itself requires a passcode. This ensures the protection of the data’s confidentiality and integrity.

### Confidentiality {27}

The data collected in the study will be handled and stored in accordance with the Research Data Management Policy of Karolinska Institutet and Stockholm County and in compliance with both Swedish and European legislation. All study personnel involved in the trial are obligated to uphold confidentiality principles in their capacity as healthcare system employees.

### Plans for collection, laboratory evaluation, and storage of biological specimens for genetic or molecular analysis in this trial/future use {33}

See above 26b there will be no biological specimens collected.

### Statistical methods

#### Statistical methods for primary and secondary outcomes {20a}

The data analytic plan is uploaded in a separate file at the OSF https://osf.io/7qsb3. The data analyses will be carried out by an independent statistician who is not part of the research group. This statistician will also remain blind to the group allocation throughout the duration of the trial.

#### Interim analyses {21b}

No interim analysis is planned within this trial.

#### Methods for additional analyses (e.g., subgroup analyses) {20b}

To detect differences in treatment effects by gender, we will analyze the interaction between treatment arm and gender. Between- and within-group moderation tests will also be performed based on baseline characteristics such as PTSD and complex PTSD symptom severity, type of trauma, comorbidity, level of threat during the traumatic event, educational level, occupational status, comorbidity factors, disturbances in self-organization, perceived working alliance, and treatment credibility. Mediators of treatment outcome to be considered are avoidance behavior and negative cognitions on the CAPS-5 and PCL-5 and change in emotional responding (SUD-ratings).

#### Methods in analysis to handle protocol nonadherence and any statistical methods to handle missing data {20c}

All available data will be used in the models. Screening, recruitment, consent forms, withdrawal, amendments to protocol and occurrence of protocol deviations, and serious adverse events will be monitored by A + Science. In case of detected irregularities, additional on-site monitor visits will be scheduled.

### Plans to give access to the full protocol, participant-level data, and statistical code {31c}

Participants will provide consent to share deidentified information for future research. Deidentified datasets can be obtained from the principal investigator upon reasonable request and under formal data transfer agreements to facilitate additional research. Moreover, transparency and reproducibility will be upheld by making the study protocol and the statistical code utilized accessible to interested parties.

### Oversight and monitoring

#### Composition of the coordinating center and trial steering committee {5d}

The team responsible for recruitment and administrative task of the daily tasks in the trial meets weekly. The group consists of the principal investigator, project leader, and staff responsible for recruitment activities. A + Science will monitor the trial, such as the presence of signed informed consent, documentation of inclusion/exclusion criteria, and randomization for each participant, and compare the digital data with source data. A minimum of 14 visits to the study site are planned during the study period.

#### Composition of the data monitoring committee, its role and reporting structure {21a}

The intervention researched in the trial is of low risk, and the Human Research Ethics Committee does not require a Data Monitoring Committee.

### Adverse event reporting and harms {22}

Therapists and assessors are instructed to immediately notify the principal investigator study team should they be concerned that a participant has caused, or is likely to cause, significant harm to themselves or others and complete a risk assessment. If a participant scores more than 2 points on the self-reported PHQ-9 item 9, which assesses the presence and duration of suicidal ideation, they are identified through an alert flag in the digital platform and subsequently contacted by a clinician via phone.

The occurrence of undesirable treatment effects can be reported by the participant at any point through the study period by alerting the designated therapist or assessor or contacting the study personnel. The therapist and assessor will be instructed to ask follow-up questions about the intensity, duration, and characteristics of the adverse event. All reported adverse events will be classified as mild, moderate, or severe and handled according to the clinics’ routines, reported to A + Science and described on a case-by-case basis. The event will be classified as serious if it is life-threatening, results in death or persistent/significant disability/incapacity, requires hospitalization or its prolongation, or is considered medically significant by the principal investigator. The NEQ will be used posttreatment and follow-ups to capture potential adverse effects in a structural way. Previous studies that have investigated intensive prolonged exposure have not detected any serious adverse events. All participants will be closely monitored during the study, and additional treatment will be provided in case of an unlikely severe deterioration.

### Frequency and plans for auditing trial conduct {23}

A + Science will perform regular audits during the trial. Potential changes in the protocol that arise will be described in detail on the Open Science Framework (OSF) and Clinicaltrials.org, and if needed, an amendment will be submitted to the Swedish Ethical Review Authority.

### Plans for communicating important protocol amendments to relevant parties (e.g., trial participants, ethical committees) {25}

If needed, amendments to the Swedish Ethical Review Authority will be submitted.

### Dissemination plans {31a}

Participants in a previous pilot study (manuscript in preparation) on intensive treatment were interviewed about their experiences, and some modifications were made to the treatment protocol accordingly. The findings in the trial will have important clinical implications for the treatment of PTSD and are of relevance to afflicted individuals, policy makers, clinicians, stakeholders, media, and the public. The results will be published in open access, scientific journals and by presenting the results at several national and international key conferences. The results will further be communicated more widely with help from the communication department at Karolinska Institutet and Region Stockholm. Since regular health care is already involved in the project, implementation is expected to be straightforward.

## Discussion

The study is the first trial to directly compare an intensive treatment for PTSD with first-line individual PE delivered for 15 weekly sessions in a Swedish psychiatric setting. The study is well powered as a superiority trial. If iPE are superior or comparable in terms of clinical efficacy and come with a lower dropout rate as well as faster recovery, it should be considered a candidate for implementation in regular care.

### Strengths of the study

The research group is highly experienced in trauma and PTSD research and in conducting randomized controlled trials. The therapists and assessors used in the trial received thorough training and supervision in the treatments investigated and the clinical gold-standard measure used to assess PTSD, respectively. Recruitment conducted in a Swedish psychiatric setting will provide valuable insights that can be applicable to routine psychiatric outpatient care.

### Challenges

The trial will be conducted in a publicly funded psychiatric outpatient setting in Stockholm, Sweden. From an implementation perspective, that is a great advantage. The major possible problem we can identify is therapist retention. The therapists delivering the treatment are employed by the county and typically have high caseloads to start with. Taking part in the trial is an additional responsibility that could be perceived as burdensome.

If a therapist or an assessor needs to terminate their engagement in the study, it can take some time first to find a replacer and train them in the treatment and in the CAPS-5.

The study also has some limitations. Therapists and participants will not be blinded to the allocated treatment arm. Blinded assessors will be used, and routines to improve blinding integrity will be developed specifically for this trial. Several measures to increase the overall scientific trustworthiness of the trial have been taken. The study hypotheses and the data analytic plan were prospectively registered. The chosen outcome measures for the study are considered the gold standard.

## Trial status

Inclusion started 12th of September 2023 and is expected to end in September 2025. The last follow-up appointment is expected to take place in October 2026. Data analysis and reporting of results will begin when all data from the primary endpoint have been collected (October 2025). Date and version identifier of the current study protocol: 2024–02-16 v 2.0.

## Data Availability

The principal investigator Bragesjö will serve as the data custodian. Deidentified datasets can be obtained from the principal investigator upon reasonable request and under formal data transfer agreements to facilitate additional research.

## Abbreviations

AQoL-6D: Assessing Quality of Life 6 Dimensions
CAPS-5: Clinician-Administered PTSD Scale for DSM-5
CBT: Cognitive behavioral therapy
CEQ: Credibility/Expectancy Questionnaire
CHEERS: Consolidated Health Economic Evaluation Reporting Standards
CONSORT: Consolidated Standards of Reporting Trials
DSM-5: Diagnostic and Statistical Manual of Mental Disorders, fifth edition
GCP: Good Clinical Practice
IPE: Intensive prolonged exposure
ITQ: The International Trauma Questionnaire
NEQ: Negative Effects Questionnaire
RCT: Randomized controlled trial
SUD: Subjective Units of Distress Scale
TIC-P: Treatment Inventory of Costs in Psychiatric Patients
PHQ-9: Patient Health Questionnaire
PTSD: Post-traumatic stress disorder
WAI-SR: Working Alliance Inventory–Short Form Revised
